# Atherosclerosis Can Be Mitochondrial: A Review

**DOI:** 10.7759/cureus.6987

**Published:** 2020-02-13

**Authors:** Josef Finsterer

**Affiliations:** 1 Neurology, Krankenanstalt Rudolfstiftung, Vienna, AUT

**Keywords:** atherosclerosis, arteriosclerosis, mitochondrial, mtdna, oxidative phosphorylation, respiratory chain, vasculopathy

## Abstract

One of the systems that are potentially affected in mitochondrial disorders, but hardly get systematically investigated, are the arteries. One of the phenotypic manifestations in arteries is atherosclerosis. This review focuses on the current knowledge and recent advances of mitochondrial atherosclerosis. We conducted a systematic literature review via PubMed using appropriate search terms.

Atherosclerosis in mitochondrial disorders may result from a primary pathomechanism or a secondary one due to mitochondrial diabetes, arterial hypertension, or hyperlipidemia. Anecdotal reports show that primary atherosclerosis can be a phenotypic feature of mitochondrial disorders. Predominantly, patients carrying mutations in mtDNA-located genes may develop primary mitochondrial atherosclerosis. Though not systematically investigated, it is conceivable that primary mitochondrial atherosclerosis results from increased oxidative stress, mitophagy, metabolic breakdown, or lactic acidosis. Mitochondrial disorder patients with primary mitochondrial atherosclerosis should receive not only antithrombotic medication but also antioxidants and cofactors.

Atherosclerosis in mitochondrial disorders may occur even in the absence of classical atherosclerosis risk factors, suggesting that atherosclerosis can be a primary manifestation of the metabolic defect. Though primary atherosclerosis in mitochondrial disorders has not been systematically investigated, anecdotal data indicate that mitochondrial dysfunction can be a mechanism for the development of primary, mitochondrial atherosclerosis. These patients require antioxidants and cofactors in addition to antithrombotic medication.

## Introduction and background

Since the first recognition of mitochondrial disorders (MIDs), it had been noticed that they sooner or later become multisystem diseases, affecting all organs or tissues, including the arteries [1]. Affection of the arteries manifests either as macro-angiopathy or micro-angiopathy [1]. Clinically, mitochondrial microangiopathy manifests as leukoencephalopathy, migraine-like headache, or peripheral retinopathy [1]. Mitochondrial macroangiopathy manifests clinically as ectasia of arteries, aneurysm formation, dissection, spontaneous arterial rupture, or atherosclerosis (ASCL) [1]. This systematic review focuses on the current knowledge and recent advances of mitochondrial ASCL. Its scientific content comprises the confirmation that mitochondrial atherosclerosis exists, that the absence of classical risk factors for ASCL does not exclude it, and that patients with mitochondrial ASCL should receive antioxidants and cofactors in addition to anti-thrombotic management.

## Review

Method

A PubMed search with the search terms “mtDNA”, “mitochondrial disorder”, “mutation”, “respiratory chain”, and “MELAS”, combined with “atherosclerosis”, “angiopathy”, “endothel”, “plaque”, “atheroma”, “vascular smooth muscle cells”, and “arteriopathy”, was carried out. After screening the abstracts, articles in English that reported patients or animals with a mitochondrial disorder (MID) and manifested with ASCL, which could not be explained by the presence of classical risk factors, such as diabetes, arterial hypertension, smoking, hyperlipidemia, or infection with C-reactive protein (CRP) elevation (primary mitochondrial ASCL), were included. In addition, experimental studies of animal models or cell cultures were considered. Included were also reviews about mitochondrial vasculopathy and studies on secondary mitochondrial ASCL. Excluded were editorials, letters, or articles written in non-English idioms.

Results

We screened 263 abstracts to find out if they met the inclusion/exclusion criteria. Lastly, 29 articles were included. Evidence for the existence of primary mitochondrial ASCL comes from several case reports, case studies, and experimental investigations. In addition, ASCL has been reported in association with several mtDNA variants.

Primary Mitochondrial ASCL

Several patients with an MID have been reported in whom ASCL was one among other clinical manifestations. In a 38-year-old male with mitochondrial encephalopathy, lactic acidosis, and stroke-like episodes (MELAS) syndrome due to the variant m.3243A>G in tRNA(Leu), magnetic resonance angiography (MRA) and digital subtraction angiography revealed a distal stenosis of the right internal carotid artery (ICA) and sparse cortical vessels in the absence of any classical risk factor [2]. Additionally, this patient had migraine since age 18 years, hearing impairment, and recurrent stroke-like episodes (SLEs) [2]. The patient was initially put on aspirin and statins but did not improve. On the contrary, he profited from coenzyme-Q (CoQ), L-arginine, vitamin-C, and valproic acid [2]. In another case report, a 58-year-old female with a six-year history of cognitive decline was diagnosed with early-onset, subcortical, ischemic, vascular dementia in the absence of any classical risk factors for ASCL [3]. She also did not have atrial fibrillation. Investigations for cardiac disease were normal but she carried the mtDNA variant m.3316G>A in ND1 [3]. In a further case report, a 57-year-old female with pigmentary retinopathy, epilepsy, ataxia, and neuropathy due to the mtDNA variant m.8839G>C in ATP6 with acute ischemic stroke in the territory of the right median cerebral artery (MCA) was reported [4]. Workup for the causes of stroke disclosed any cardiac cause, particularly heart failure and atrial fibrillation, and conventional angiography was normal. Classical risk factors for ASCL were also absent [4]. In a seven-year-old female with MELAS due to the variant m.3243A>G in tRNA(Leu) (heteroplasmy 58%) and the variant c.985A>G in medium-chain acyl-CoA dehydrogenase (MCAD), MRA revealed occlusion of the left ICA in the absence of atrial fibrillation and heart failure or any classical risk factor for ASCL [5]. With the progression of the disease, the right ICA also became involved, resulting in the development of Moya Moya syndrome [5]. In a Chinese study of patients with mitochondrial encephalomyopathy, ICA stenosis was found in two patients and posterior cerebral artery (PCA) stenosis in one patient. Unfortunately, the study is not accessible via established databanks and only cited in a study by Sun et al. (2018) [2]. In a nine-year-old male with MELAS due to the variant m.3243A>G in tRNA (Leu), MRA revealed a segmental narrowing in the crural segment of the right PCA with good distal runoff [6]. The patient did not carry any classical risk factors for ASCL. The patient was treated with dichloroacetate, dexamethasone, thiamine, and CoQ [6]. In a seven-year-old female with MELAS, conventional cerebral angiography revealed an occlusion of the right PCA in the absence of any risk factors for ASCL [7]. In a 21-year-old male with MELAS/Kearns-Sayre overlap syndrome and Wolff-Parkinson-White (WPW) syndrome, cerebral angiography revealed irregularities and, respectively, occlusions of several branches of the left anterior cerebral artery (ACA) and MCA [8]. In a recent study on patients with methyl-malonic aciduria/encephalopathy, an increased prevalence of lacunar stroke in the globus pallidus bilaterally was found, suggesting that the metabolic defect is associated with the development of cerebral microangiopathy [9].

In a study of 225 patients with myocardial infarction (MI), a positive correlation between MI and the variant m.5178C>A was found [10]. A negative correlation was reported between MI and the variants m.14846G>A and m.12315G>A [10]. It was concluded that the variant m.5178C>A represents a risk factor for the development of MI and that the variants m.14846G>A and m.12315G>A may have a protective effect for MI [10]. In a study of 65 patients with coronary heart disease (CHD) and 23 atherosclerotic plaques, it was found that an mtDNA deletion of 4977 bp was present in 26.2% of the patients as compared to 4.5% in controls [11]. Also, the heteroplasmy rates of the deletion were higher in patients as compared to controls, ranging from 18% to 46% [11]. The heteroplasmy rates were neither influenced by classical risk factors for ASCL nor by any clinical parameter [11]. In a recent study on endothelial cells from patients with MELAS due to the variant m.3243A>G, it was found that endothelial cells were diseased and found to be pro-atherogenic and pro-inflammatory due to high levels of reactive oxygen species (ROS), OxLDLs, and a high basal expression of vascular cell adhesion molecule-1 (VCAM-1), particularly isoform-b [12]. In dysfunctional endothelial cells, more monocytes adhered to endothelial cells than in control cells, suggesting an atherosclerosis-like pathology in MELAS [12]. Notably, these pathological findings could be reversed by treatment with antioxidants, suggesting that lowering ROS is crucial for MELAS patients [12]. In a study by Tanaka et al. (2004), the mtDNA variant m.8794T>C in ATP6 was associated with coronary sclerosis [13]. Unfortunately, the original paper was not accessible and only the description by Blanco-Grau was available [4]. In a study of patients with CHD, it has been shown that the amount of mtDNA in leukocytes is reduced [14]. It was concluded that the amount of leukocyte mtDNA predicts the severity of coronary atherosclerosis [14]. In a study of 482 patients with CHD, the variant m.16189T>C was associated with an increased risk of CHD [15]. Also in a Saudi cohort of 669 patients, the polymorphism m.16189T>C was associated with an increased risk of CHD [16]. Furthermore, there are indications that certain haplogroups (e.g. haplogroup-T) predispose for the development of ASCL [17]. In a Russian study, an association between the variants m.652delG, m.3336C>T, m.12315G>A, m.14459G>A, and m.15059G>A with the development of carotid atherosclerosis was found [18].

Secondary Mitochondrial ASCL

In 2009, Iizuka et al. reported a 40-year-old male with short stature, mental retardation, hypoacusis, diabetes, and cataract, who developed recurrent ischemic strokes in the territory of the right MCA in the absence of any other ASCL risk factors than diabetes [19]. At age 35 years, the patient experienced a cortical infarct in the right MCA territory with complete remission of weakness and dysarthria [19]. At age 36 years, he experienced an occlusion of the right ICA, MCA, and ACA [19]. Despite treatment with argatroban and intravenous heparin, he recovered only with deficits. At age 37 years, he experienced a third ischemic stroke, again due to occlusion of the right MCA [19]. Except for the right ICA, ASCL was found in no other vascular territory. His HbA1c values were 8.0 respectively 7.7 [19]. Genetic workup revealed the variant m.617G>A in tRNA(Phe) [19]. In a 41-year-old male with MELAS due to the variant m.3243A>G, diabetes, and kidney cancer, histological workup of the resected kidney revealed widespread interstitial fibrosis and prominent vascular lesions, with the vessel displaying marked intimal fibrosis, and arterioles with hyaline deposits typical of hyaline arteriolosclerosis [20]. In an autopsy study of two brothers with hypoacusis, diabetes, progressive photomyoclonic epilepsy, and progressive renal insufficiency, after death in their thirties, biochemical investigations of the muscle revealed a combined complex-III and complex-IV defect [21]. Histological workup in skin fibroblasts, kidney, and liver revealed premature, systemic ASCL and arteriolosclerosis [21]. A 50-year-old male with chronic progressive external ophthalmoplegia (CPEO), diabetes, and CHD required coronary bypass surgery for CHD [22]. Muscle and myocardial biopsy showed ragged-red fibers and glycogen deposits suggesting MID, which was confirmed after a 5019 bp deletion of the mtDNA had been detected [22].

Discussion

This review shows that there are strong indications for premature ASCL as a primary and secondary manifestation of an MID. Primary and secondary ASCL in MIDs is a risk factor for cerebrovascular events. Primary and secondary mitochondrial ASCL require, in addition to anti-thrombotic treatment, antioxidants or cofactors. Whether primary mitochondrial ASCL contributes to the pathogenesis of small lymphocytic lymphomas (SLLs) remains elusive but is rather unlikely given the fact that SLLs are not confined to a vascular territory and are characterized by hyperperfusion rather than hypoperfusion on perfusion-weighted imaging (PWI) and single-photon emission tomography (SPECT) in the acute stage [23]. The review also shows that in patients with a multisystem MID, ASCL with a consecutive occlusion of arteries and arterioles may occur in a single territory without affecting other territories.

Explanations for the development of primary ASCL in MIDs is oxidative stress, mitophagy, metabolic breakdown, and lactic acidosis. Increased oxidative stress explains the pathogenesis of primary ASCL, as it leads to lipid peroxidation, damage of mitochondrial DNA (mtDNA), mitochondrial dysfunction, damage of endothelial cells, vascular smooth muscle cells (VSMC), erythrocytes, thrombocytes, and, lastly, to apoptosis via either the receptor-mediated pathway or the mitochondrion-mediated pathway and the activation of the caspase cascade [24]. A primary defect of the respiratory chain or oxidative phosphorylation may be associated with reduced energy output and, lastly, a break down of the energy metabolism. Uncoupling of the electron transfer from ATP synthesis results in the excess generation of ROS, leading to widespread cellular injury and cardiovascular disease [25]. Regarding ASCL as a chronic inflammatory disease, it is essential to embank this process. A mechanism that contributes to inflammation is mitophagy [26]. Accordingly, if mitophagy is impaired, the inflammatory process may spread and result in focal or generalized ASCL [26]. In a study of heteroplasmy rates and mtDNA copy number of autopsy-derived samples of aortic intima differing by the type of atherosclerotic lesions, it was found that heteroplasmic mtDNA mutations are characteristic of particular areas of intimal tissue [27]. In 83 intimal samples, 55 heteroplasmic variants were found; the mean minor allele frequencies level accounted for 0.09, with a 12% mean heteroplasmy level [27]. The variance of the mtDNA copy number measured in adjacent areas of the intima was high, but atherosclerotic lesions and unaffected intima did not differ with regard to the mtDNA copy number [27]. The authors concluded that heteroplasmic mtDNA variants correspond to the extent of atherosclerotic phenotypes [27]. In another study, it has been shown that ROS activated by post-prandial triglyceride lipolysis (TGRL) products (TL) increases the expression of the stress-responsive protein, activating transcription factor-3 (ATF-3), which injures human brain microvascular endothelial cells in vitro [28]. In a further study, it has been shown that ATF3-T4 predominantly regulates the TL-induced brain microvascular inflammation and TNF signaling [29]. Both siRNAs of ATF3-T4 and ATF3-T5 suppressed cell apoptosis and lipotoxic brain microvascular endothelial cells [29]. The authors concluded that signaling pathways, triggered by the ROS-responsive transcript variants ATF3-T4 and ATF3-T5, contribute to cerebral microvascular inflammation induced by TL [29].

Primary ASCL in MIDs needs to be differentiated from secondary ASCL in MIDs. Secondary ASCL in MIDs is more frequent than primary ASCL and results from mitochondrial diabetes, mitochondrial arterial hypertension, hyperlipidemia, or smoking [30-31]. Mitochondrial hypertension has been particularly reported in Chinese patients [31]. Whether these patients also experience more frequently secondary ASCL has not been systematically investigated yet. Primary and secondary ASCL may coexist. Mitochondrial diabetes particularly occurs in mitochondrial syndromes, such as MELAS, KSS, and MIDD [31]. Whether hyperlipidemia is truly a primary manifestation of an MID is under debate, but there are indications that it may represent a mechanism to compensate for impaired glucose metabolism.

Whether the spontaneous rupture of arteries in MIDs is due to atherosclerosis or due to "weakness" of the arterial wall is under debate, but it is conceivable that both mechanisms are involved in the pathogenesis of spontaneous ruptures [32]. Unfortunately, autopsy reports do not mention whether there was ASCL at the site of the rupture [32]. Whether dissection of arteries in MID patients results from focal ASCL or from a weakness of the arterial wall remains speculative. Since ASCL has not been found at the site of the dissection, experts favor that dissection in MIDs rather results from a weakness of the arterial wall than from ASCL [33]. That dissection of arteries in MID patients rather results from a “weakness” of the arterial wall than from ASCL has been shown in a patient with MELAS due to the variant m.3243A>G, who experienced carotid and vertebral artery dissection and showed marked vascular abnormalities on histological examination of the skin and muscle [34].

Evidence for ASCL as a primary manifestation of MIDs

Table 1 lists the evidence for ASCL as a primary manifestation of MIDs based on our literature search.

**Table 1:** Evidence for ASCL as a primary manifestation of MIDs ASCL: atherosclerosis, MCA: median cerebral artery, MID: mitochondrial disorder, KSS: Kearns-Sayre syndrome, tRNA: transfer ribonucleic acid, ND1: subunit 1 of respiratory chain complex-I, bp: basepair, del: deletion, A. adenine, G: guanine, C: cytosine, Leu: leucine, Phe: phenylalanine, ATP: adenosine-triphosphate, Np: not provided, PCA: posterior cerebral artery, ACA: anterior cerebral artery, ICA: internal carotid artery, PA: primary ASCL, SA: secondary ASCL

Mutated gene	Mutation	ASCL Manifestations	ASCL type	Reference
tRNA(Leu)	m.3243A>G	Carotid artery stenosis	PA	[2]
ND1	m.3316G>A	Ischemic, vascular dementia	PA	[3]
Multiple	Multiple	Carotid ASCL	PA	[10]
ATP6	m.8839G>C	MCA	PA	[4]
tRNA(Phe)	m.617G>A	Recurrent ICA, MCA, ACA occlusion	SA	[19]
Np	5019 bp del	Coronary heart disease	SA	[22]
tRNA(Leu)	m.3243A>G	Carotid artery occlusion	PA	[5]
Np	Np	Carotid artery stenosis (n=2), PCA stenosis (n=1)	PA	[2]
Multiple	4977 bp del	Coronary artery disease	PA	[11]
tRNA(Leu)	m.3243A>G	PCA stenosis	PA	[6]
ATP6	m.8794T>C	Coronary sclerosis	PA	[13]
Np (MELAS)	Np	PCA occlusion	PA	[7]
Np	Np	Premature ASCL	SA	[21]
Np (MELAS/KSS)	Np	Occluded ACA + MCA branches	PA	[8]
tRNA(Leu)	m.3243A>G	Renal arteriolosclerosis	SA	[20]

## Conclusions

Though primary and secondary ASCL in MIDs have not been systematically investigated, anecdotal data currently available indicate that mitochondrial dysfunction can be a mechanism for the development of ASCL. ASCL in MIDs may be prevalent even in the absence of classical ASCL risk factors, suggesting that ASCL can also be a primary manifestation of the metabolic defect. Mechanisms explaining the development of primary ASCL in MIDs could be oxidative stress, mitophagy, metabolic dysfunction, or lactic acidosis. MID patients with primary ASCL should carefully monitor their risk factors for ASCL to avoid the additional development of secondary ASCL. MID patients with primary or secondary ASCL should receive antioxidants and cofactors in addition to anti-thrombotic medication.
